# The SLC25A45-TML Axis as a Biological Foundation for a Multivariable Plasma Metabolite Signature for High-Precision Prostate Cancer Detection

**DOI:** 10.3390/cancers18101571

**Published:** 2026-05-12

**Authors:** Liang Zhao, Raghothama Chaerkady, Naseruddin Höti, Eric Zhao, Anirudh Kashyap, Morgan Fair, Qing Wang, Xiaonan Kang

**Affiliations:** 1 Complete Omics Inc., Baltimore, MD 21227, USA; 2 Gilman School, Baltimore, MD 21210, USA; 3 Complete Omics (Hangzhou) Inc., Hangzhou 311200, China; 4 Renji Hospital, School of Medicine, Shanghai Jiao Tong University, Shanghai 200127, China

**Keywords:** SLC25A45-TML axis, metabolomics, prostate cancer diagnosis, fatty acid oxidation, polyamine metabolism, bipartite metabolic ratios

## Abstract

Background: Prostate cancer remains a significant global health burden, yet current diagnostic reliance on PSA screening is heavily hampered by limited specificity and high rates of overdiagnosis. Methods: To address this clinical bottleneck, we utilized a highly sensitive Complete360^®^-MyMeta targeted-metabolomics platform to perform high-resolution profiling of 43 metabolites across the carnitine, polyamine, and methylation networks in plasma from a discovery cohort of all-stage (I–IV) PCa patients and healthy controls. Results: Our analysis identified 28 significantly altered metabolites (*p* < 0.05), revealing profound systemic metabolic reprogramming characterized by the depletion of circulating TML and putrescine, alongside the elevation of L-acetylcarnitine and sarcosine. These systemic shifts are consistent with a localized tumoral “metabolic sink”, wherein upregulated mitochondrial TML import via the SLC25A45 transporter actively fuels fatty acid oxidation, while parallel androgen signaling drives massive polyamine synthesis. Translating these mechanistic insights into a clinical tool, we developed a multivariable diagnostic signature utilizing mathematically stable bipartite metabolic ratios. An optimized, cross-validated model combining L-acetylcarnitine/TML and sarcosine/putrescine effectively mitigated physiological noise to achieve robust diagnostic separation, yielding an area under the curve (AUC) of 0.99. Conclusions: Ultimately, this study provides a discovery-phase proof-of-concept for the SLC25A45-TML axis as a mechanistically grounded, stage-independent liquid biopsy, offering a rational, non-invasive framework to significantly improve PCa detection.

## Simple Summary

Current blood tests for prostate cancer (PCa), such as the standard primarily prostate-specific antigen (PSA) screen, often lack the specificity needed to accurately detect the disease, frequently leading to overdiagnosis and unnecessary biopsies. To address this clinical challenge, we utilized a highly sensitive analytical platform, Complete360^®^-MyMeta, to search for new, more reliable chemical markers in patient blood. Our findings indicate that as prostate tumors grow, they act as a localized “metabolic sink”, actively pulling specific circulating nutrients—particularly trimethyllysine (TML) and polyamine precursors—out of the bloodstream to fuel their massive energy demands. By simultaneously measuring the depletion of these circulating nutrients and the elevation of their metabolic byproducts, we developed a new diagnostic signature based on mathematical ratios. In our initial discovery cohort, this ratio-based approach successfully minimized natural physiological background noise, achieving highly accurate detection of prostate cancer across all clinical stages. Ultimately, this work provides a strong biological foundation for a new, non-invasive “liquid biopsy”. Pending confirmation in larger clinical trials, this mechanistically grounded test could significantly improve early detection and reduce the burden of unnecessary medical procedures for patients.

## 1. Introduction

Prostate cancer remains a preeminent global health challenge, ranking as the second most frequently diagnosed malignancy and a leading cause of cancer-related mortality among men worldwide [1,2,3]. For decades, clinical management has relied heavily on prostate-specific antigen screening. However, the inherent lack of specificity in PSA testing—often confounded by benign prostatic hyperplasia (BPH) and prostatitis—has precipitated a crisis of overdiagnosis and subsequent overtreatment [4,5,6,7,8]. With up to 50% of patients undergoing unnecessary invasive biopsies for indolent disease, there is an urgent mandate for the discovery of non-invasive biomarkers that possess not only superior diagnostic precision but also a rigorous biological foundation [9,10,11].

A defining hallmark of PCa oncogenesis is its distinct metabolic reprogramming. Unlike many solid tumors that exhibit the classic Warburg effect, PCa cells demonstrate a unique metabolic plasticity characterized by a heightened dependence on fatty acid oxidation (FAO) and mitochondrial oxidative phosphorylation for energy production [12,13,14,15]. As the malignancy progresses, the bioenergetic and biosynthetic demands of the tumor escalate exponentially. To sustain rapid proliferation, particularly within the often nutrient-deprived tumor microenvironment, PCa cells exploit lipids as their primary and most efficient carbon source. This metabolic pivot requires a massive, sustained upregulation of mitochondrial activity. For FAO to proceed, long-chain fatty acids must be continuously imported into the mitochondrial matrix, where they undergo β-oxidation to generate copious amounts of acetyl-CoA. This influx subsequently fuels the tricarboxylic acid (TCA) cycle, driving high-capacity ATP generation via oxidative phosphorylation [16,17]. Consequently, the tumor’s survival becomes fundamentally tethered to the efficiency of its mitochondrial import mechanisms.

To sustain this high-capacity FAO phenotype, PCa cells require a continuous supply of carnitine, the obligate cofactor for shuttling long-chain fatty acids across the inner mitochondrial membrane via the carnitine palmitoyltransferase system [18,19]. Consequently, the metabolic gatekeepers governing carnitine availability represent a critical, yet underexplored, vulnerability in prostate cancer biology. Recent functional studies by Dias et al. (2025) have identified the mitochondrial transporter SLC25A45 as exactly such a gatekeeper, demonstrating its essential role in the intracellular import of TML—the rate-limiting precursor for de novo carnitine biosynthesis [20]. We hypothesize that advancing PCa tumors aggressively upregulate SLC25A45 to sequester systemic TML, thereby creating a “metabolic sink” to fuel continuous carnitine production and sustain their heightened energetic demands. Crucially, this localized tumoral sequestration should manifest as a detectable, continuous depletion of TML within the systemic circulation, transforming a fundamental mechanism of intratumoral FAO activity into a non-invasive diagnostic window.

Beyond lipid metabolism, PCa is characterized by significant dysregulation in polyamine synthesis and methylation pathways, both of which are under the tight control of androgen receptor (AR) signaling [21,22,23]. AR signaling is known to drive a massive anabolic demand for polyamines, such as putrescine and spermidine, which are essential for DNA stabilization, protein synthesis, and cellular proliferation [24]. Furthermore, elevated methyltransferase activity—reflected in altered levels of sarcosine and modified nucleosides—serves as a functional readout of the epigenetic turnover driving PCa progression [25,26].

The pivotal role of this hyper-active methylation in PCa progression was definitively established by Sreekumar et al., who identified sarcosine—an N-methyl derivative of the amino acid glycine—as a critical differential metabolite [25]. Through unbiased global metabolomic profiling of prostate tissues and biofluids, they revealed that sarcosine levels are highly elevated during the transition from clinically localized prostate cancer to metastatic disease and can even be detected non-invasively in urine. This accumulation of sarcosine is intrinsically linked to a strong enrichment of methyltransferase activity, including the upregulation of the histone methyltransferase EZH2, which is frequently observed in metastatic tumors. Crucially, the “sarcosine story” illustrates that hyper-active methylation pathways produce active mediators of tumorigenesis rather than just passive metabolic byproducts. Sreekumar et al. demonstrated that the addition of exogenous sarcosine, or the experimental knockdown of the sarcosine-degrading enzyme sarcosine dehydrogenase (SARDH), directly induced an invasive phenotype in benign prostate epithelial cells. Conversely, attenuating the expression of glycine-N-methyltransferase (GNMT)—the enzyme responsible for generating sarcosine from glycine—effectively reduced intracellular sarcosine levels and hampered prostate cancer cell invasion. This mechanistic evidence, coupled with the observation that androgen receptor and ERG gene fusions coordinately regulate components of the sarcosine pathway, underscores how the synergistic dysregulation of polyamine and methylation networks actively drives the aggressive cellular phenotypes required for PCa metastasis.

Among the various “omics” technologies, metabolomics is uniquely positioned to address the urgent clinical need for novel, non-invasive PCa biomarkers. Because metabolites represent the ultimate downstream products of genomic, transcriptomic, and proteomic regulation, the metabolome provides a direct, real-time biochemical snapshot of the tumor’s actual phenotypic state [27,28]. By capturing the end-products of the aforementioned lipid metabolism, polyamine metabolism, and methylation dysregulation, metabolomics offers the unparalleled promise of translating complex intracellular oncogenic signaling into measurable, systemic signatures in biofluids such as plasma [29,30]. However, translating this biological promise into robust clinical applications has proven challenging. Despite the promise of metabolomics in capturing these complex shifts, previous biomarker discovery efforts have often been hindered by analytical variability, limited sensitivity for low-abundance species, and a lack of mechanistic interpretability that links metabolite alterations to specific oncogenic processes. Historically, the field has relied heavily on untargeted profiling, which frequently identifies statistically significant but biologically arbitrary panels of metabolites. These isolated, single-metabolite biomarkers are highly susceptible to physiological noise—fluctuating dramatically due to diet, systemic inflammation, or renal clearance—leading to high rates of failure during external validation. Furthermore, untargeted platforms often suffer from dynamic range limitations, masking critical, low-abundance tumor intermediates beneath highly abundant systemic metabolites [31,32,33].

Ultimately, the overarching bottleneck in clinical metabolomics is the reliance on empirical correlation over biological causation. To overcome the limitations of systemic noise and analytical variability, next-generation biomarker discovery must shift toward targeted, hypothesis-driven approaches. By focusing on mathematically stable metabolite ratios that reflect specific, rate-limiting enzymatic or transport bottlenecks—such as those governed by AR signaling or mitochondrial importation—we can anchor diagnostic signatures in definitive, experimentally validated tumor biology rather than transient statistical associations. To overcome the analytical limitations of untargeted profiling, this study leverages a targeted UHPLC-QqQ-MS/MS workflow to perform high-resolution quantification of critical metabolites encompassing the SLC25A45-associated TML axis, polyamine synthesis, and methylation networks. This sensitive platform enabled the reliable detection of low-abundance biological intermediates across clinical stages I–IV. Here, we report the development of a multivariable diagnostic signature based on mathematically stable bipartite metabolic ratios—specifically L-acetylcarnitine/TML and sarcosine/putrescine. We demonstrate that this ratio-based approach effectively mitigates systemic physiological noise and magnifies the pathological signal by directly coupling opposing, mechanistically linked metabolic fluxes. Within our discovery-phase cohort (*n* = 70), a rigorously cross-validated linear model combining these ratios achieved robust diagnostic separation (AUC = 0.998), demonstrating the potential to significantly improve upon the baseline predictive value of traditional PSA screening (historical AUC 0.58–0.70) [34,35]. Ultimately, these findings establish a discovery-phase proof-of-concept for a mechanistically grounded liquid biopsy. By translating the complex intracellular rewiring of PCa—specifically the tumor’s function as a localized “metabolic sink”—into measurable systemic readouts, we provide a rational diagnostic framework that warrants rigorous evaluation in large, independent clinical validation trials.

## 2. Materials and Methods

### 2.1. Clinical and Pathological Details of Prostate Cancer Patients

All participants recruited in this study include patients with histologically confirmed prostate cancer (PCa, *n* = 35) across all clinical stages (I–IV) and healthy normal controls (HC, *n* = 35). The plasma samples were obtained from the commercial vendor Innovative Research (Novi, MI, USA), and sample collection was approved by an Institutional Review Board or Independent Ethical Committee, and patients gave their informed consent. Demographic and clinical characteristics of patients with PCa are given in Table 1.

### 2.2. Sample Collection and Preparation

All samples were collected following standardized clinical fasting protocols to minimize dietary metabolic interference. Plasma was separated via centrifugation at 4 °C and immediately stored at −80 °C until metabolomic analysis to preserve the integrity of labile metabolites. For metabolite extraction and analysis, plasma samples were placed on ice and allowed to thaw slowly. Then, 250 μL cold methanol (stored at −20 °C) was added to 50 μL of the plasma sample. The mixture was vortexed, followed by incubation at −20 °C for 6–8 h to precipitate the proteins. The metabolite-containing supernatant was transferred to a new 1.5 mL Eppendorf tube (Eppendorf North America, Enfield, CT, USA) after centrifugation at 15,000 rpm at 4 °C for 10 min. The extracted samples were dried under nitrogen gas and reconstituted in 150 μL 50% methanol:water (*v*/*v*) solution. After a brief vortex and one-hour incubation on a shaker at 4 °C, the reconstituted samples were clarified for 10 min at 15,000 rpm. The final supernatants were transferred to 300 μL sample vials for further LC-MS analysis.

### 2.3. Sample Quality Control and Lifecycle Metadata Management

Because the entire workflow from clinical sample collection to omics data generation carries potential quality risks, a comprehensive quality management framework was implemented to ensure analytical robustness and traceability in this study. The framework follows the principles outlined in the standards “Minimum Data Set Specification for Clinical Plasma Proteomics Sample Quality” and “Minimum Data Set Specification for Clinical Plasma Metabolomics Sample Quality”, which provide standardized guidance for plasma-based omics studies. The quality management system includes four key components. (i) Sample quality characterization: Lifecycle metadata were recorded for each specimen, including blood collection time, centrifugation delay, storage duration, number of freeze–thaw cycles, and hemolysis index. (ii) Sample quality grading: Samples were categorized into four quality levels (Grade I–IV) based on the recorded parameters to guide appropriate matching between sample quality and study objectives. (iii) Method performance verification: Precision assessments, system suitability evaluation, and QC sample monitoring were embedded throughout the analytical sequence to continuously assess assay stability and reproducibility. (iv) Data quality traceability: Key sample-quality parameters and batch information were incorporated into downstream statistical analyses to enable identification, evaluation, and correction of potential technical bias. Through this closed-loop quality management framework, the present study achieved full lifecycle traceability from sample acquisition to omics data generation, ensuring robust biomarker discovery and supporting downstream clinical translation.

### 2.4. Metabolic Profiling

Metabolic profiling was performed using the Complete360^®^-MyMeta platform (Complete Omics Inc., Baltimore, MD, USA) as previously described [36]. Briefly, in order to achieve the superior sensitivity and ensure the detection of low-abundance biomarkers critical for early-stage diagnosis, an optimized targeted LC-MS method comprising 43 candidate metabolites was established, and the platform is operated on an ultra-high-performance liquid chromatography coupled to tandem mass spectrometry (UHPLC-MS/MS) using an Agilent UHPLC system interfaced with an Agilent 6495C QqQ mass spectrometer (Agilent Technologies, Santa Clara, CA, USA). Samples (5 µL) were injected and separated on a Waters BEH Amide column (2.1 mm × 100 mm, 1.7 µm) with a flow rate of 0.25 mL/min. Mobile phases consisted of (A) water with 10 mM ammonium formate and 0.1% formic acid and (B) acetonitrile with 10 mM ammonium formate and 0.1% formic acid. The gradient program was: 0–1 min, 98% B; 1–10.5 min, 98–60% B; 10.5–13 min, 60–15% B; 13–13.5 min, 15% B; and 13.5–14 min, 15–98% B with a post-time of 6 min for column equilibration. The column compartment temperature was maintained at 30 °C. The Agilent 6495 triple quadrupole with Jet Stream ion source was operated in positive/negative ion switching mode using multiple reaction monitoring (MRM) with optimized transitions and source parameters: gas temp: 250 °C; gas flow rate: 12 L/min; nebulizer: 35 psi; sheath gas temp: 350 °C; sheath gas flow: 12 L/min; capillary voltage: 3000 V (positive), 2500 V (negative). The acquired mass spectrometry data were qualitatively and quantitatively analyzed by Agilent MassHunter Qualitative Analysis and Quantitative Analysis software package (version: B.10.00).

### 2.5. Statistical Analysis and Modeling

Statistical analysis and biomarker performance evaluation were performed separately with “Statistical Analysis”, “Pathway Analysis”, and “Biomarker Analysis” modules (R-based packages, version 4.2.0) under the MetaboAnalyst 6.0 framework (http://www.metaboanalyst.ca/MetaboAnalyst/, accessed on 18 February 2026) [37]. Pre-processed data for both PCa and HC samples were submitted to this web-based tool to perform fold change analysis, two-sample t-tests, and Wilcoxon rank-sum tests. Significant features were defined by a threshold of (fold change ≥ 1.2) and (*p*-value < 0.05). Statistical significance for individual metabolites was determined using a strict False Discovery Rate (FDR) < 0.01. A fold-change (FC) threshold of ≥1.2 was applied. While higher thresholds (e.g., FC ≥ 1.5) are common in tissue-level analyses, a 1.2 threshold was specifically selected for this plasma cohort to account for the strict homeostatic buffering and dilution effects inherent to circulating biofluids, ensuring that biologically significant systemic metabolic shifts were not erroneously discarded as false negatives. Biomarker identification and performance evaluation were performed sequentially with classical univariate receiver operating characteristic (ROC) curve analysis and multivariate analysis. The corresponding area under the curve (AUC) values and 95% confidence intervals (CI) were generated using 500 bootstrap iterations. Specifically, multivariate ROC curve analyses are performed based on three multivariate algorithms: linear-support vector machines (SVM), random forests, and partial least squares discriminant analysis (PLS-DA).

## 3. Results

### 3.1. Plasma Metabolomic Profiling Identifies Altered Metabolisms in Prostate Cancer

**Figure 1:**
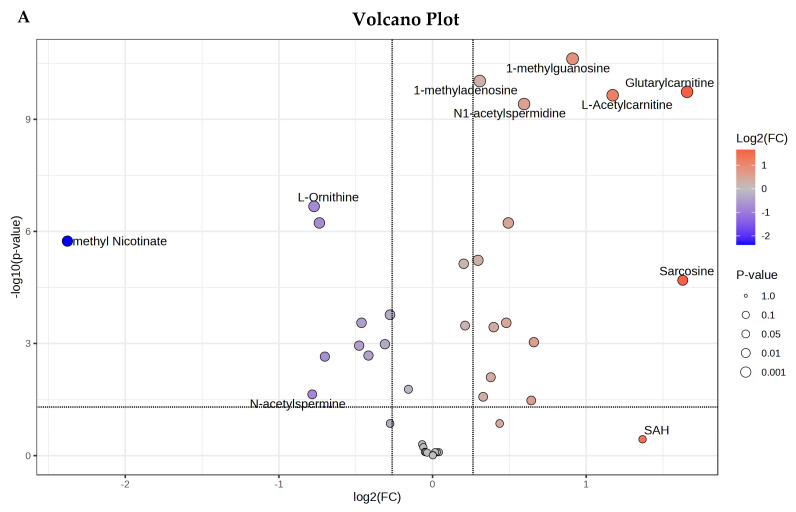

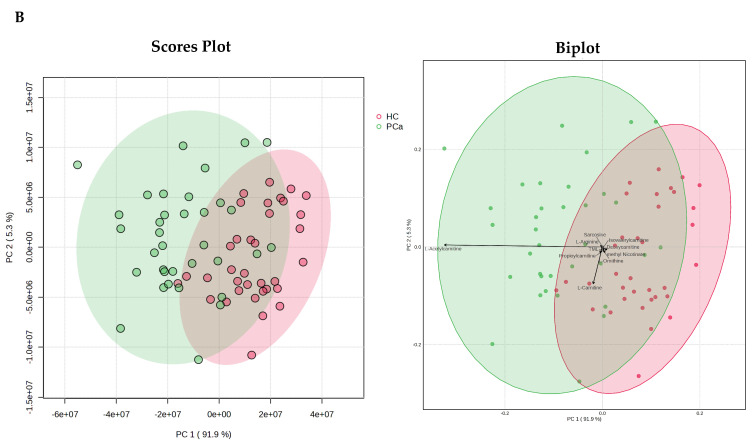

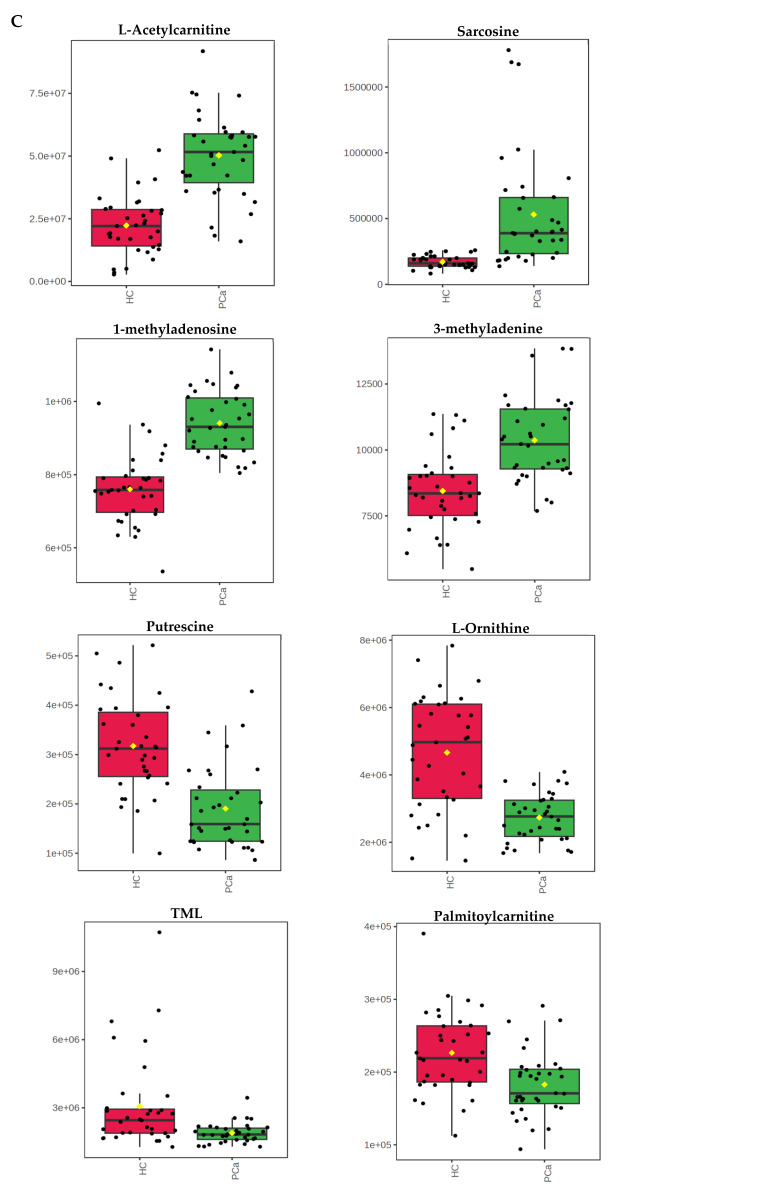

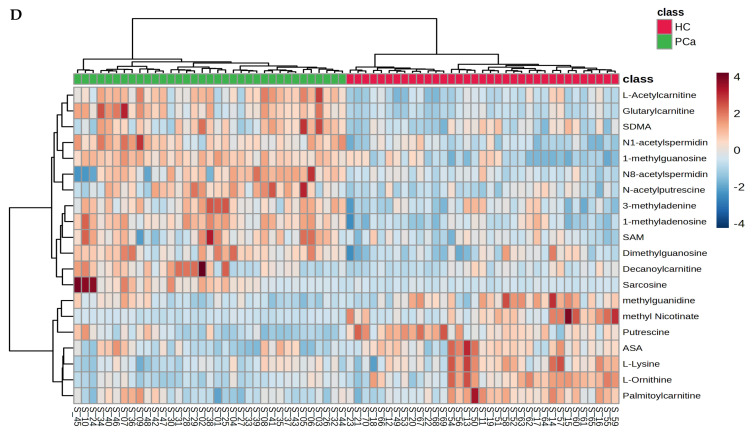
Human plasma from prostate cancer patients shows dramatically disturbed metabolic profiles. (**A**) Volcano plot illustrating the most significantly altered metabolites in the plasma of patients with prostate cancer (PCa). (**B**) PCA score plot (**left**) showing the first and second principal components and separation of the patients with and without PCa. The Biplot (**right**) simultaneously illustrates sample (shown as dots) clusters and the driving metabolites (shown as vectors/arrows) using the top two principal components. The direction of the vector shows the metabolite’s contribution to the PCs, with long vectors indicating a strong influence. (**C**) The relative quantitation of the representative significant metabolites detected in human PCa plasma vs. healthy plasma. L-acetylcarntine, sarcosine, and two methylated metabolites (1-mthyladenosine and 3-methyladenine) were observed at elevated levels in PCa, while putrescine, L-ornithine, TML, and palmitoylcarnitine were observed at lower levels in PCa, suggesting that PCa causes distinct metabolic profiles in plasma. The black dots in the boxplot represent the concentrations of the selected metabolite from all samples. The notch indicates the 95% confidence interval around the median of each group, defined as ±1.58 × IQR/sqrt(*n*). The mean concentration of each group is indicated with a yellow diamond. (**D**) Heatmap of the top 20 metabolites that were statistically significantly different in PCa compared to normal plasma, which reflects the metabolic reprogramming in PCa, including fatty acid oxidation, methylation metabolism, and polyamine metabolism. Abbreviations: TML = trimethyllysine, SAM = S-adenosine methionine, SDMA = symmetric dimethylarginine, ASA = argininosuccinic acid.

To characterize the metabolic landscape of prostate cancer (PCa), we performed targeted metabolomics on plasma from PCa patients and healthy controls (HC). As shown in the volcano plot (Figure 1A), we identified 28 significantly altered metabolites (with an adjusted *p*-value < 0.05, see Supplementary Table S1 for a detailed list). Of these, 17 metabolites were significantly upregulated, and 11 were significantly downregulated. Principal component analysis (PCA) demonstrated a clear separation between the PCa and HC cohorts (Figure 1B, left), with the biplot confirming that specific metabolites from the carnitine and polyamine pathways drive this divergence (Figure 1B, right). In particular, L-acetylcarnitine was identified as the metabolite that has the strongest influence. Relative quantitation of some of the most significant metabolites detected in human PCa plasma vs. HC plasma was illustrated in Figure 1C. Specifically, we observed significant elevations in L-acetylcarnitine, sarcosine, 1-methyladenosine, and 3-methyladenine in PCa plasma. Conversely, metabolites such as putrescine, L-ornithine, TML, and palmitoylcarnitine exhibited significant depletion in PCa compared to healthy controls. A heatmap of the top 20 statistically different metabolites (Figure 1D) highlighted key trends. Mapping these differential metabolites to their respective biochemical pathways, we observed a total of seven metabolites (SDMA, 1-methyladenosine, 3-methyladenine, 1-methyladenosine, SAM, dimethylguanosine, and sarcosine) involved in methylation metabolism, which were all upregulated in PCa plasma. In addition, the pathway analysis based on these 28 significantly changed metabolites revealed that the activity of carnitine metabolism, as well as the polyamine metabolism, was disturbed in PCa. Of novel biomarker discovery interest was the fact that we also identified a metabolite, TML, that exhibits a consistent biological depletion in PCa plasma samples compared to healthy controls, which makes it a potential biomarker candidate. TML is a methylated basic amino acid, and it functions as a precursor for de novo carnitine synthesis and plays key roles in fatty acid oxidation and energy metabolism in different types of tumors. Our metabolomic analysis reflects the distinct metabolic reprogramming across fatty acid oxidation, methylation, and polyamine metabolic axes between PCa and HC.

### 3.2. Discovery of Candidate Biomarkers for All-Stage PCa Plasma

After evaluation of the metabolomic profiles of PCa vs. HC plasma, we identified a subset of four metabolites (including L-acetylcarnitine, sarcosine, putrescine, and TML) that demonstrated robust differences across two classes and could potentially serve as biomarkers for PCa diagnosis. Among these four differential metabolites, L-acetylcarnitine and sarcosine demonstrated elevated levels (2.25-fold and 3.09-fold, respectively), while putrescine and TML had diminished levels (0.60-fold and 0.61-fold, respectively) in PCa compared to HC plasma. At least one metabolite from each of the above three disturbed metabolic pathways in PCa was selected to reflect the important roles they play during cancer development and progression. In a previous study, sarcosine, an N-methyl derivative of glycine, was identified as a differential metabolite that was highly increased in prostate cancer and may have potential as a promising biomarker for PCa. Our findings in this study about the upregulation of sarcosine and other methylated metabolites were consistent with this trend and supported the increased methyltransferase activities during prostate cancer progression. Next, we applied both univariate and multivariate models with different algorithms to explore the potential of these four metabolites as candidates for the development of biomarker panels for PCa detection.

### 3.3. Univariate Diagnostic Performance

**Figure 2:**
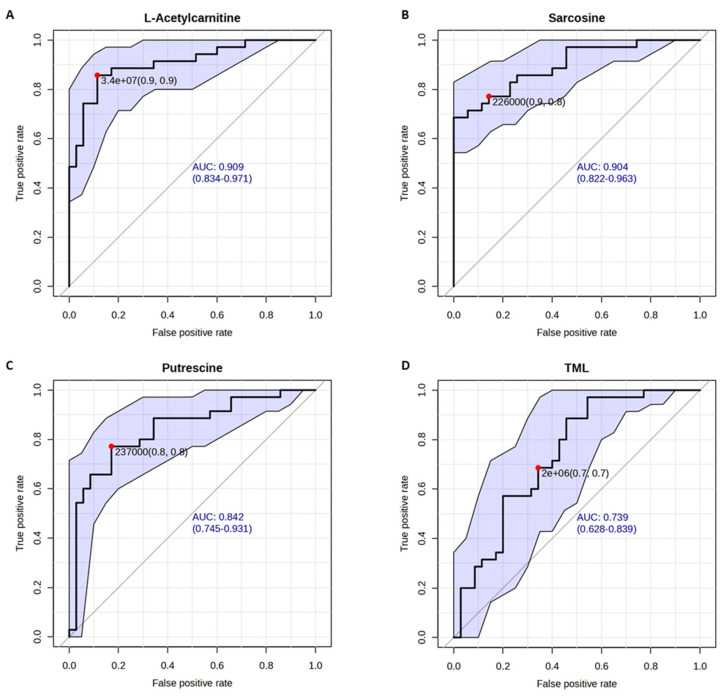
Univariate ROC curve analyses for all-stage PCa vs. HC. Calculated AUC scores as well as the 95% confidence intervals (blue shade area) for selected metabolite biomarkers: (**A**) L-acetylcarnitine (AUC = 0.909; 95% CI, 0.834–0.971); (**B**) sarcosine (AUC = 0.904; 95% CI, 0.822–0.963); (**C**) putrescine (AUC = 0.842; 95% CI, 0.745–0.931); and (**D**) TML (AUC = 0.739; 95% CI, 0.628–0.839). The red dot data in each ROC curve represent the computed cutoff value for the selected biomarker with the corresponding sensitivity and specificity.

For univariate analysis, we first assessed whether our selected four biomarkers could discriminate between PCa cases and HC. The overall receiver operating characteristic curves (ROCs) for these four biomarkers are displayed in Figure 2. L-acetylcarnitine performed the best, with all-stage PCa versus HC yielding an area under the curve (AUC) of 0.909 (95% CI, 0.834–0.971; Figure 2A), which indicates its predictive value is already good as an individual biomarker. Sarcosine performed similarly to L-acetycarnitine, yielding an AUC of 0.904 (95% CI, 0.822–0.963). For the remaining two biomarkers, an AUC of 0.842 (95% CI, 0.745–0.931) was achieved for putrescine, and an AUC of 0.739 (95% CI, 0.628–0.839) was achieved for TML. The fact that an AUC of 1.0 indicates perfect prediction and an AUC of 0.5 indicates a prediction equivalent to random selection. Thus, all four selected biomarkers hold the potential to discriminate between PCa and HC.

### 3.4. Multivariate Diagnostic Performance

**Figure 3:**
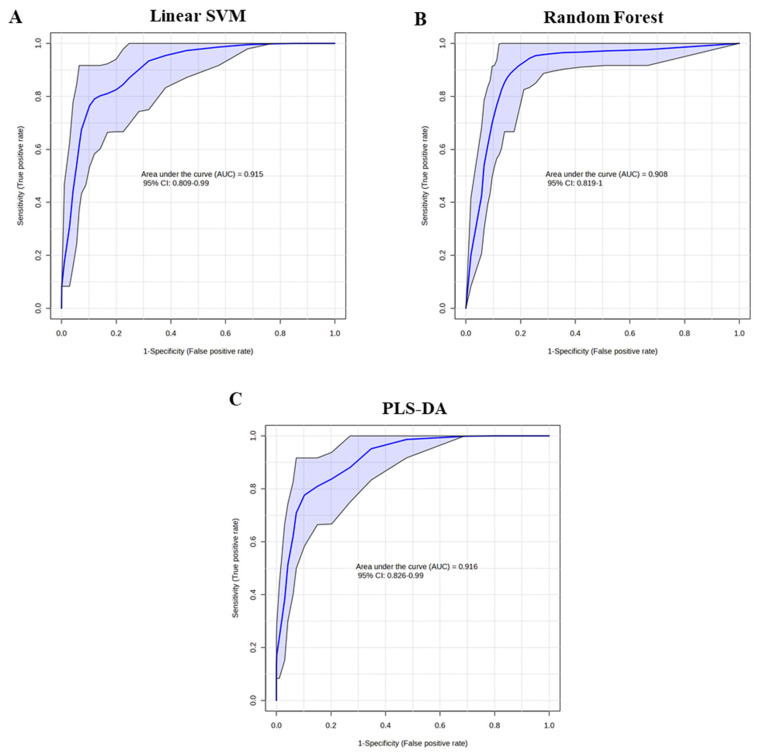
Two-feature (L-acetylcarnitine and putrescine) ROC curve analyses for all-stage PCa vs. HC based on three different algorithms. (**A**) Linear-support vector machines (SVM): (AUC = 0.915; 95% CI, 0.809–0.99). (**B**) Random Forests: (AUC = 0.908; 95% CI, 0.819–1). (**C**) Partial least squares discriminant analysis (PLS-DA): (AUC = 0.916; 95% CI, 0.826–0.99). The blue shade area in each ROC curve represents the computed 95% confidence band.

To move beyond univariate screening, we developed an optimized multivariable model. We prioritized features based on their variable Importance in Projection (VIP) scores and individual AUC performance. We identified a core signature consisting of four metabolites: sarcosine, putrescine, L-acetylcarnitine, and TML. As an example to demonstrate the improved performance that can be achieved with multivariate models, we selected a two-feature panel (L-acetylcarnitine and putrescine) using three different machine learning algorithms (Figure 3) to evaluate the performance. All three two-feature models performed better than any single biomarker alone. When comparing all-stage PCa versus HC, the following AUCs were observed after analysis: The linear-support vector machines (SVMs) model achieved an AUC of 0.915 (95% CI, 0.809–0.99), while Random Forest and partial least squares discriminant analysis (PLS-DA) yielded AUCs of 0.908 (95% CI, 0.819–1) and 0.916 (95% CI, 0.826–0.99), respectively.

**Figure 4:**
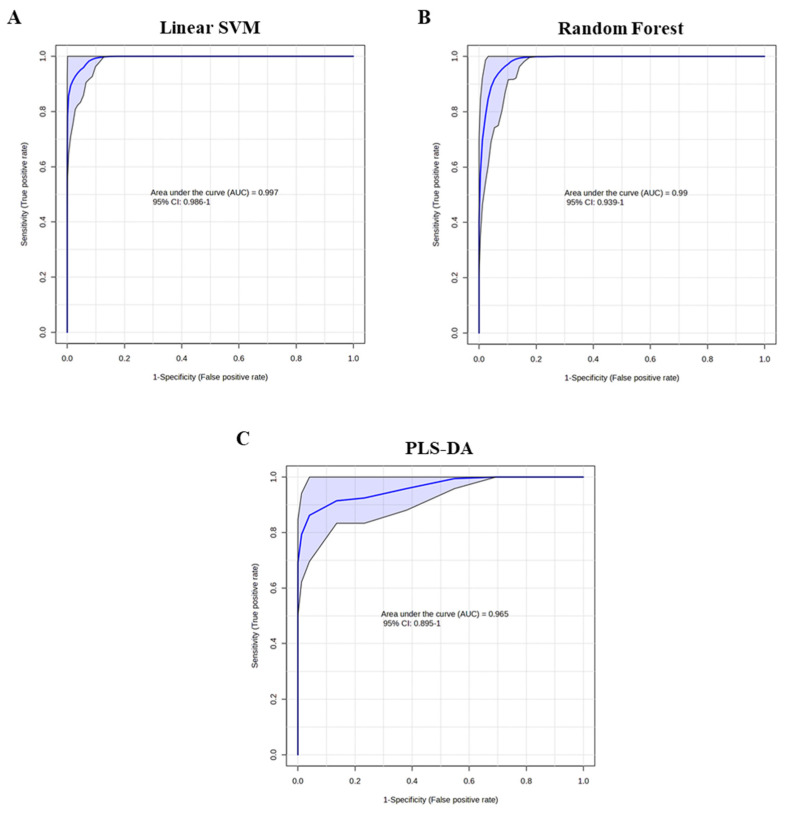
Multivariate (L-acetylcarnitine, putrescine, sarcosine, and TML) ROC curve analyses for all-stage PCa vs. HC based on three different algorithms. (**A**) Linear-support vector machines (SVM): (AUC = 0.997; 95% CI, 0.986–1). (**B**) Random Forests: (AUC = 0.99; 95% CI, 0.939–1). (**C**) Partial least squares discriminant analysis (PLS-DA): (AUC = 0.965; 95% CI, 0.895–1). The blue shade area in each ROC curve represents the computed 95% confidence band.

Finally, a consolidated four-marker panel (a combination of L-acetylcarnitine, sarcosine, putrescine, and TML) was evaluated across these algorithms (Figure 4). The multivariable models demonstrated further improvement over univariate and two-feature models. Notably, the best performing model with the linear SVM algorithm yielded an AUC of 0.997 (95% CI, 0.986–1), superior performance for all-stage PCa detection, while the multivariate model with the Random Forest algorithm showed a comparable AUC of 0.99 (95% CI, 0.939–1), and the model with the PLS-DA algorithm yielded a less improved AUCs of 0.965 (95% CI, 0.895–1).

### 3.5. Using Bipartite Metabolite Ratios to Improve Diagnostic Performance for PCa

**Figure 5:**
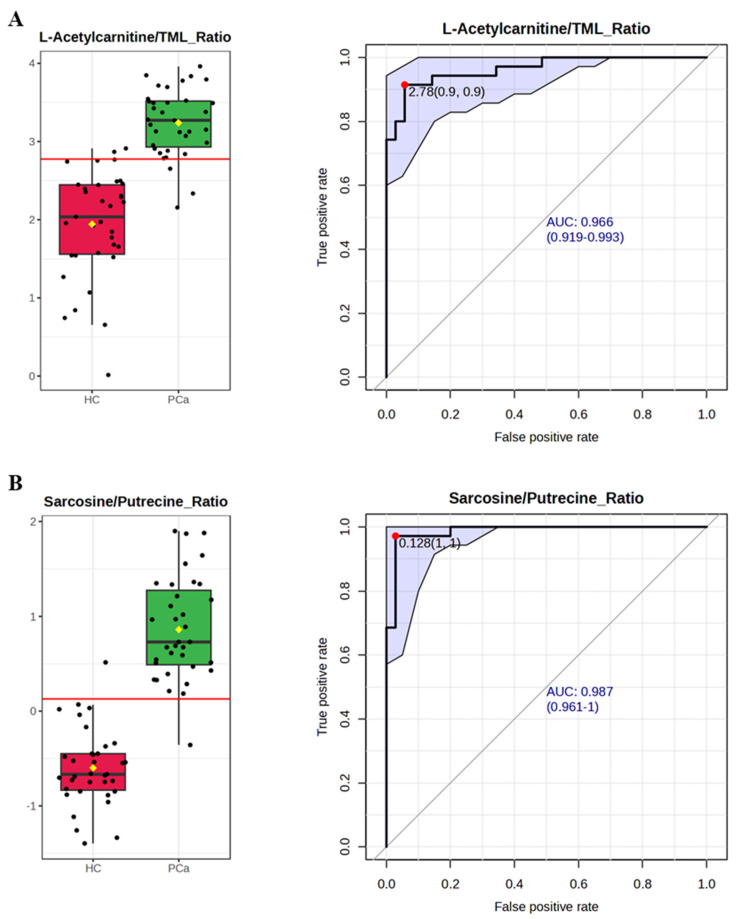

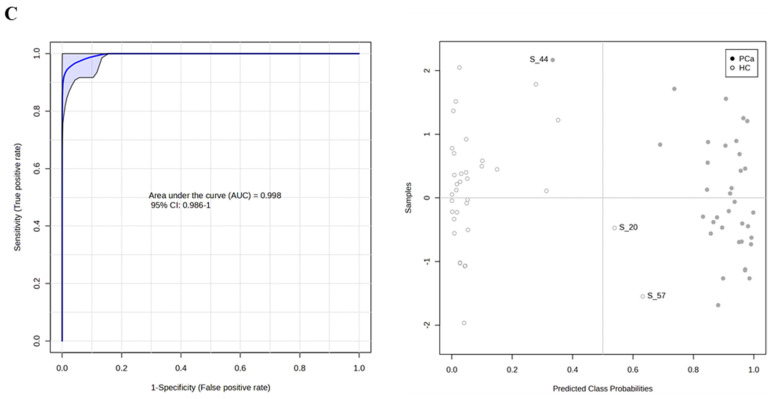
Diagnostic performance and multivariate analysis of the bipartite metabolite signature for all-stage PCa vs. HC. (**A**) L-acetylcarnitine/TML ratio: Box plots showing the relative levels based on the specific bipartite metabolite signature in human PCa vs. HC plasma. ROC curve showing the corresponding diagnostic performance (AUC = 0.966; 95% CI, 0.919–0.993). (**B**) Sarcosine/putrescine ratio: Box plots showing the relative levels based on the specific bipartite metabolite signature in human PCa vs. HC plasma. ROC curve showing the corresponding diagnostic performance (AUC = 0.987; 95% CI, 0.961–1). The black dots in the boxplot represent the concentrations of the selected metabolite from all samples. The notch indicates the 95% confidence interval around the median of each group, defined as ±1.58 × IQR/sqrt(*n*). The mean concentration of each group is indicated with a yellow diamond. The optimal cutoff is indicated with a horizontal red line on the boxplot. (**C**) Multivariate (ratio combination of L-acetylcarnitine/TML and sarcosine/putrescine, linear SVM model) analysis: ROC curve showing the improved diagnostic performance (AUC = 0.998; 95% CI, 0.986–1). The predicted class probabilities across 100 cross-validations illustrate a clear separation between PCa and HC samples (labeled samples were classified to the wrong groups; the classification boundary is located at the center, and x = 0.5). The blue shade area in each ROC curve represents the computed 95% confidence band.

To amplify the biological signal and reduce inter-patient variability, we converted these key metabolic features into two strategic bipartite ratios: L-acetylcarnitine/TML and sarcosine/putrescine. These ratios combine metabolites with opposing regulatory patterns in the tumor microenvironment, mathematically compounding the divergence between PCa and healthy states to provide improved diagnostic sensitivity and specificity. To establish a baseline for their individual diagnostic power, we first performed an independent evaluation of the two bipartite ratios. As shown in Figure 5A,B, both ratios demonstrated robust diagnostic potential in distinguishing PCa patients from healthy controls (HC) when analyzed separately. Independent ROC analysis of the L-acetylcarnitine/TML ratio yielded a high AUC of 0.966 (95% CI: 0.919–0.993). The sarcosine/putrescine ratio exhibited even stronger individual discriminatory power, achieving an independent AUC of 0.987 (95% CI: 0.961–1.0).

To maximize overall diagnostic accuracy, we integrated these two complementary ratios into a multivariate classification model. The combined bipartite signature demonstrated superior performance, achieving very good discrimination between all-stage PCa and HC with an optimized AUC of 0.998 (95% CI: 0.986–1.0) (Figure 5C, left). Further analysis of the predicted class probabilities (Figure 5C, right) through cross-validation confirmed the model’s robustness, revealing a distinct and strict separation boundary between the PCa and HC sample distributions. Based on its exceptional predictive accuracy and stability, this multivariable ratio model (linear SVM model) was selected as the optimal signature for our future large-cohort validation studies.

To further characterize the clinical utility of our bipartite metabolic signature, we conducted a subgroup analysis evaluating its capacity to differentiate between high-grade (Gleason score 8–10; *n* = 21) and low-grade (Gleason score ≤ 7; *n* = 14) prostate cancer. ROC curve analysis revealed that the two primary diagnostic ratios utilized in our multivariable model did not exhibit significant discriminatory power for histological grading; the L-acetylcarnitine/TML and sarcosine/putrescine ratios yielded AUCs of 0.575 (95% CI: 0.384–0.793) and 0.498 (95% CI: 0.303–0.696), respectively (Figure S1A,B). Ultimately, the inability of the core bipartite signature to stratify patients by Gleason score supports its function as a grade-independent diagnostic biomarker. The systemic depletion of critical precursors driven by the tumoral “metabolic sink” appears to be a fundamental, early-stage physiological shift that remains consistently detectable across the spectrum of tumor aggressiveness.

Having established that the “metabolic sink” profile is a grade-independent physiological shift, we next sought to evaluate the clinical utility of our platform for early detection. To do this, we conducted a targeted subgroup analysis assessing the diagnostic performance of the bipartite metabolic signature within the early-stage, low-grade patient cohort (Gleason score ≤ 7; *n* = 14) against the healthy control group (*n* = 35). ROC curve analysis demonstrated that the bipartite ratios maintain robust discriminatory power even in the earliest stages of clinically detectable disease. Specifically, the L-acetylcarnitine/TML ratio achieved an AUC of 0.959 (95% CI: 0.882–0.998), while the sarcosine/putrescine ratio yielded an AUC of 0.994 (95% CI: 0.969–1.000) (Figure S2A,B). These predictive values confirm that the systemic sequestration of TML and polyamine precursors, alongside the elevation of their downstream metabolites, is not merely a late-stage consequence of advanced tumor burden. Instead, this signature acts as a sensitive, early indicator of oncogenic metabolic reprogramming, highlighting its substantial potential as a non-invasive screening tool for the early detection of prostate cancer.

## 4. Discussion

The development of a new diagnostic platform with enhanced specificity and accuracy is essential to address the clinical limitations of PSA screening, thereby reducing the burden of unnecessary biopsies and the overtreatment of indolent disease. Through the application of ultra-sensitive LC-MS-based metabolomics, we identified a distinct metabolic signature that discriminates prostate cancer patients from healthy controls with high precision.

The metabolic profiles visualized in Figure 1 underscore profound systemic disturbances associated with malignancy. The clear clustering in PCA space and the specific metabolic trajectories identified in our biplot analysis suggest that PCa induces a state of systemic metabolic reprogramming rather than isolated biochemical shifts. One of the most striking findings of this study is the consistent upregulation of seven methylated metabolites in PCa plasma. Notably, sarcosine—a methyl derivative of glycine previously identified as a potential PCa biomarker—was significantly elevated in our cohort. This accumulation, along with other methylated species, reflects the hyper-active methyltransferase environment and dysregulated epigenetic turnover that serve as hallmarks of PCa oncogenesis.

**Figure 6:**
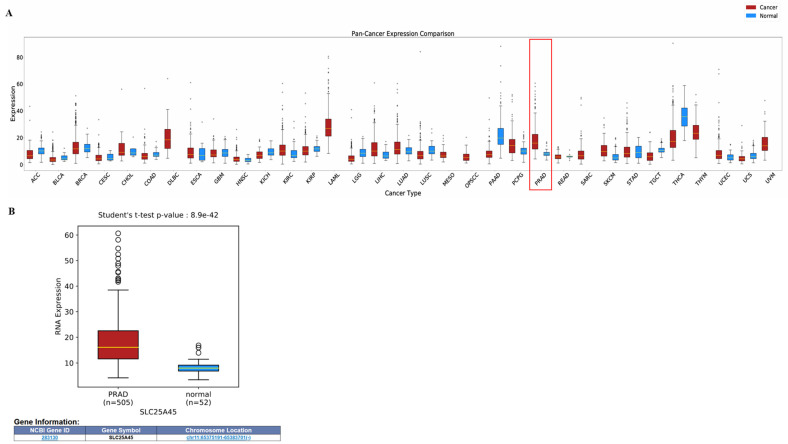
SLC25A45 is highly expressed in prostate cancer. (**A**) Gene expression levels of SLC25A45 across different types of cancers in the TCGA dataset. (**B**) Comparison of RNA expression levels of SLC25A45 between primary prostate adenocarcinoma (PRAD, *n* = 505) and adjacent normal tissues (*n* = 52). ACC: adrenocortical carcinoma; BLCA: bladder urothelial carcinoma; BRCA: breast invasive carcinoma; CESC: cervical squamous cell carcinoma and endocervical adenocarcinoma; CHOL: cholangiocarcinoma; COAD: colon adenocarcinoma; DLBC: lymphoid neoplasm diffuse large b-cell lymphoma; ESCA: esophageal carcinoma; GBM: glioblastoma multiforme; HNSC: head and neck squamous cell carcinoma; KICH: kidney chromophobe; KIRC: kidney renal clear cell carcinoma; KIRP: kidney renal papillary cell carcinoma; LAML: acute myeloid leukemia; LGG: brain lower grade glioma; LIHC: liver hepatocellular carcinoma; LUAD: lung adenocarcinoma; LUSC: lung squamous cell carcinoma; MESO: mesothelioma; OPSCC: oropharyngeal squamous cell carcinoma; PAAD: pancreatic adenocarcinoma; PCPG: pheochromocytoma and paraganglioma; READ: rectum adenocarcinoma; SARC: sarcoma; SKCM: skin cutaneous melanoma; STAD: stomach adenocarcinoma; TGCT: testicular germ cell tumors; THCA: thyroid carcinoma; THYM: thymoma; UCEC: uterine corpus endometrial carcinoma; UCS: uterine carcinosarcoma; UVM: uveal melanoma.

In stark contrast to these elevations, we identified consistent depletion of TML, a methylated derivative of lysine. TML serves as the obligate precursor for de novo carnitine synthesis, playing a vital role in mitochondrial fatty acid beta-oxidation (FAO). Unlike many solid tumors that primarily exhibit the Warburg effect, PCa relies heavily on lipid oxidation to fuel mitochondrial respiration. In human circulation, TML originates primarily from the degradation of methylated proteins; its depletion in our study suggests an aggressive sequestration by the tumor microenvironment. Recent landmark discoveries have identified the mitochondrial solute carrier SLC25A45 as the transporter responsible for importing TML into the mitochondria, effectively controlling carnitine biosynthesis and metabolic fuel switching [20]. While dietary sources provide carnitine, endogenous synthesis becomes a critical bottleneck during periods of rapid proliferation or nutrient stress. To validate the clinical relevance of this axis, we analyzed SLC25A45 expression across various tissues using the Cancer Genome Atlas (TCGA) dataset (Figure 6A). Our analysis revealed that while SLC25A45 is upregulated in several malignancies, prostate adenocarcinoma exhibited the most significant overexpression (*p* = 8.9 × 10^−42^, Figure 6B). This indicates that PCa cells specifically upregulate SLC25A45 to enhance mitochondrial TML import, sustaining high-flux FAO. The observed depletion of plasma TML likely reflects this enhanced uptake from the circulation to meet the tumor’s bioenergetic demands.

While our clinical profiling demonstrates a profound systemic depletion of TML in prostate cancer patients, the mechanistic basis for this phenomenon can be understood through the “metabolic sink” hypothesis, wherein advancing tumors actively sequester circulating precursors to fuel elevated bioenergetic demands. The biological plausibility of this sink is firmly anchored by the recent functional characterization of the mitochondrial transporter SLC25A45. As definitively demonstrated by Dias et al. (2025) [20], utilizing gold-standard proteoliposome transport assays, SLC25A45 operates as a highly specific, critical gatekeeper for the mitochondrial import of methylated amino acids, notably TML. Their genetic ablation models further established that the loss of SLC25A45 severely attenuates mitochondrial TML uptake, precipitating a collapse in de novo carnitine biosynthesis. This reliance on extracellular pools is corroborated by targeted flux analyses; [^2^H_9_]-TML isotope labeling confirmed that exogenous TML is rapidly internalized and channeled through the TMLHE and BBOX1 enzymatic pathway to generate carnitine [20]. By integrating these validated functional mechanics with the widespread upregulation of SLC25A45 observed in TCGA-PRAD transcriptomic profiles, a cohesive physiological model emerges: the aggressive overexpression of SLC25A45 by prostate tumors acts as a persistent metabolic vacuum, actively driving the cellular sequestration of TML. Consequently, it is this localized, voracious intratumoral demand for carnitine biosynthesis that ultimately manifests as the detectable systemic depletion of TML captured by our non-invasive plasma signature. Corroborating this “metabolic sink” model, we also observed a decrease in long-chain acylcarnitines (such as palmitoylcarnitine and stearoylcarnitine) in plasma, reinforcing the paradigm of PCa’s unique dependence on lipid consumption. Conversely, the elevation of L-acetylcarnitine may reflect intense intracellular FAO activity, where excess acetyl groups are exported back into circulation as a secondary energy source. To our knowledge, this is the first study to propose a TML-based biomarker panel for PCa diagnosis, providing a mechanistically grounded alternative to traditional markers.

A further central finding is the significant depletion of polyamine pathway metabolites, including L-ornithine and putrescine, in PCa plasma. This observation is highly consistent with the metabolic vulnerabilities identified in the recent literature, where androgen receptor (AR) signaling drives a massive anabolic demand for polyamines. By upregulating Ornithine Decarboxylase (ODC1), the tumor creates a high-flux “sink” that voraciously sequesters systemic ornithine and putrescine to support the synthesis of spermidine and spermine, which are essential for DNA stabilization and proliferation.

The observation that a localized tumor can significantly deplete a circulating systemic metabolite raises important questions regarding whole-body mass balance and biological plausibility. A localized prostate tumor lacks the static mass to sequester the entire circulating pool of TML. Therefore, we propose that the “metabolic sink” is driven by continuous dynamic flux rather than static accumulation. TML is a specialized, low-abundance intermediate, primarily derived from endogenous protein methylation rather than direct dietary intake. In healthy physiology, the liver and kidneys are the primary consumers of circulating TML for de novo carnitine synthesis. However, by highly upregulating the SLC25A45 transporter, prostate cancer cells establish an unregulated, secondary site of synthesis. As demonstrated by recent [^2^H9]-TML isotope tracing and flux analyses, SLC25A45 facilitates the rapid, unidirectional conversion of TML into carnitine to fuel fatty acid oxidation. This continuous consumption prevents TML equilibrium, creating a persistent systemic drain that ultimately manifests as the measurable depletion observed in our clinical cohort.

Our findings align with and extend the established literature on prostate cancer metabolomics. For over a decade, alterations in amino acid and polyamine pathways have been recognized as hallmarks of PCa; for instance, the elevation of sarcosine and alterations in the polyamine pathway have been previously identified as potential markers of disease progression [25,38]. However, the clinical utility of these single markers has often been limited by high biological variability. Our study addresses this by utilizing bipartite ratios, which normalize these fluctuations. Furthermore, while the reliance of PCa on fatty acid oxidation (FAO) is well-documented [13], the specific mitochondrial gatekeeper for carnitine precursors remained elusive. By identifying the SLC25A45-mediated sequestration of TML—supported by the functional transport data in Dias et al. (2025) [20]—we provide a mechanistic link between systemic metabolite depletion and the intracellular energy demands previously described in PCa metabolomics literature.

Translating these findings into clinical utility, our multivariable modeling demonstrates the superior performance of bipartite metabolic ratios—specifically L-acetylcarnitine/TML and sarcosine/putrescine. This approach offers several strategic advantages over isolated molecular features: (1) Signal magnification: By mathematically coupling opposing biological shifts (one upregulated, one downregulated), the ratio compounds the divergence between pathological and physiological states, amplifying the effect size and statistical power [39]. (2) Intrinsic normalization: Ratios function as an internal control within each specimen, mitigating the impact of global fluctuations in sample concentration and reducing the need for external spike-in standards [40]. (3) Methodological resilience: Ratio-based metrics are more robust against “noise” introduced by differing assay platforms or pre-analytical protocols, facilitating the harmonization of datasets across multi-center studies [41,42]. (4) Superior discriminatory power: From a clinical perspective, these composite indices yield higher AUC values, improving the ability to accurately differentiate all-stage PCa from healthy controls [43]. (5) Mechanistic insight: Ratios provide a window into the homeostatic balance between competing biochemical pathways—such as the balance between carnitine uptake and FAO output—that absolute concentration levels alone may fail to characterize [44]. (6) Dimensionless comparability: As unitless values, ratios allow for seamless comparison across disparate clinical trials regardless of the specific measurement scales or units employed [41].

It is critical to distinguish between the biological association of TML with prostate cancer, the underlying mechanism driving this change, and its ultimate clinical utility. While our initial analysis revealed a consistent systemic depletion of TML in PCa plasma—an association mechanistically explained by the SLC25A45-mediated “metabolic sink”—TML alone possesses only modest standalone diagnostic power (AUC = 0.739). This highlights a common pitfall in metabolomics: a mechanistically important metabolite does not automatically translate into a robust clinical biomarker due to baseline physiological variance. Therefore, the true clinical utility of our signature is not derived from TML in isolation but from the bipartite ratio of L-acetylcarnitine/TML. By mathematically pairing the depleted precursor with its elevated metabolic product, the ratio normalizes systemic noise and amplifies the tumor-specific metabolic signal, elevating the diagnostic accuracy to a clinically actionable level.

To maximize the translational utility of the SLC25A45–TML metabolic signature, it is essential to define its intended clinical use case. The primary objective of this diagnostic platform is broad, early-stage diagnostic screening, specifically designed to address the well-documented limitations of the current PSA standard—namely, poor specificity and the subsequent overdiagnosis of indolent disease. Our data supports this application; the primary bipartite ratios maintained robust diagnostic performance (AUC > 0.95) when evaluating low-grade, early-stage disease against healthy controls. Furthermore, the inability of the signature to stratify patients by Gleason score confirms that this systemic profile is not merely an artifact of advanced tumor burden. Instead, it indicates that the voracious nutrient sequestration driving the tumoral “metabolic sink” is a fundamental, initiating physiological shift that occurs early in oncogenesis. Consequently, we envision this targeted metabolomic assay functioning as a sensitive, non-invasive frontline screening tool.

While our study demonstrates the high diagnostic potential of the SLC25A45-TML axis and bipartite metabolic ratios, several limitations must be acknowledged to provide a balanced interpretation of the findings:

- Cohort Size and Lack of Independent Validation: The primary limitation of this study is the relatively small sample size (*n* = 70) of our discovery cohort. Although internal 5-fold cross-validation demonstrated mathematical stability for our predictive models, the exceptionally high diagnostic accuracy achieved (AUC = 0.998) is characteristic of a discovery-phase study and inherently carries the risk of overfitting. Consequently, this performance cannot guarantee an unbiased model for broader populations, and the reported diagnostic accuracy should be interpreted as the maximum potential performance within a controlled dataset. To confirm the generalizability and true clinical utility of this metabolic signature, rigorous evaluation in large, independent, multi-center validation cohorts is absolutely required.
- Clinical Control Specificity and BPH: Our healthy control group was not biopsy-confirmed to exclude benign prostatic hyperplasia (BPH). Given that BPH is prevalent in the aging population and can elevate PSA levels, future studies must include a symptomatic non-cancer control group (BPH or prostatitis). This is essential to rigorously evaluate whether the L-acetylcarnitine/TML and sarcosine/putrescine ratios can effectively distinguish malignancy from benign prostatic enlargement.
- Stratification of Advanced and Metastatic Disease: While our cohort included patients with a wide range of PSA levels, including those exceeding 100 ng/mL, the present study was not powered to establish separate diagnostic thresholds for localized versus metastatic disease. The metabolic signature of late-stage PCa may be influenced by systemic cachexia or treatment effects, and the performance of these ratios specifically for the staging of metastatic disease remains to be validated in a dedicated clinical cohort.
- Mechanistic Inference vs. Causality: A primary limitation of this study is the inferential nature of the link between the systemic “metabolic sink” observed in patient plasma and the localized intracellular activity of the SLC25A45 transporter. While our hypothesis is supported by parallel TCGA transcriptomic analyses, we acknowledge that circulating plasma metabolites are highly dynamic and can be influenced by the metabolic flux of peripheral tissues. To bridge this mechanistic gap, our biological framework heavily leverages the fundamental transport biology recently elucidated by Dias et al. (2025) [20]. While their foundational proteoliposome and isotope-tracing assays provide a robust experimental proof-of-concept for the transporter’s capacity to sequester TML, we recognize the inherent limitations of directly extrapolating these models to clinical pathophysiology. To definitively validate the complex regulatory dynamics of the SLC25A45-TML axis specifically within the prostate tumor microenvironment, dedicated functional studies are necessary. Future research must utilize a diverse panel of human prostate cancer cell lines and in vivo models to rigorously isolate this transport mechanism and comprehensively map its interactions with concurrent oncogenic signaling networks.
- Sample Size and Histological Granularity: While our initial subgroup analysis (high versus low Gleason) strongly suggests that the metabolic signature acts as a grade-independent diagnostic marker, a limitation of this discovery-phase study is the relatively small sample size, which precluded a highly granular comparison across individual ISUP grade groups. The current study was not powered to definitively rule out the existence of subtle metabolic thresholds that might differentiate specific histological sub-grades. Therefore, confirming the absolute grade-independence of this systemic profile across the entire clinical and histological spectrum remains a priority for future validation in significantly larger patient cohorts.
- Validation for Early Detection: While the intended clinical application of this assay is early-stage diagnostic screening, the capacity of the signature to perform as a frontline screening tool requires further investigation. Although our subgroup analysis yielded highly promising results (AUC > 0.95), indicating strong potential for the early detection of low-grade disease, the statistical power within this specific subset is limited. Consequently, a primary objective for future prospective research must be the definitive validation of these findings strictly within large, independent cohorts of Stage I patients evaluated against meticulously matched healthy controls.
- Metabolic and Genomic Heterogeneity: Prostate cancer is a molecularly heterogeneous disease. Our current panel does not account for differences in primary oncogenic drivers, such as MYC-driven versus AKT-driven phenotypes, which are known to utilize distinct metabolic pathways. The degree to which our signature reflects a “universal” PCa metabolic state versus a specific molecular subtype warrants further investigation.
- Pre-analytical Standardization and Clinical Scalability: A critical consideration for the clinical translation of any metabolomics-based signature is the impact of pre-analytical variability. To mitigate this and ensure high-resolution data on our UHPLC-QqQ-MS/MS platform, our study utilized a strictly standardized protocol for plasma collection and processing, which included the deliberate utilization of commercially sourced healthy control plasma. However, this rigorous standardization strategy necessitated a clinical trade-off: it precluded the acquisition of paired baseline PSA measurements for the control subjects. Consequently, a direct statistical comparison of baseline PSA levels between the prostate cancer cohort and the healthy control group could not be performed. Furthermore, while our bipartite ratios are mathematically designed to provide internal normalization and enhance robustness against analytical “noise”, the stability of these specific signatures—particularly L-acetylcarnitine/TML and sarcosine/putrescine—must still be fully characterized under a wider range of pre-analytical conditions. Future prospective, multi-center studies are therefore essential. These studies must not only establish the reliability of these diagnostic thresholds when subjected to the inherent sample-handling variations in real-world clinical settings but also capture comprehensive, paired clinical metrics to fully benchmark our metabolic signature against traditional screening parameters.
- External Validation and Clinical Benchmarking: The most significant limitation of this study is its design as a discovery-phase, proof-of-concept investigation within a relatively small, highly controlled cohort (*n* = 70). Consequently, the high diagnostic accuracy (AUC = 0.998) achieved by the cross-validated linear model must be interpreted with caution, as performance frequently attenuates in broader populations. Furthermore, this study did not evaluate the signature against established clinical nomograms or commercial diagnostic panels (e.g., PHI, 4Kscore). Therefore, while the analytical mass spectrometry workflow is compatible with future clinical laboratory standards, the signature itself is not yet ready for clinical deployment. Establishing true clinical utility and determining whether this model actively improves upon current decision-making paradigms will require rigorous evaluation in a large, independent, multicenter validation cohort.

Finally, the transition of the SLC25A45–TML metabolic signature from a research discovery to a clinical diagnostic tool necessitates a balanced evaluation of economic feasibility and practical workflow integration. From an economic standpoint, although high-resolution UHPLC-QqQ-MS/MS instrumentation requires an initial capital investment, the operational cost per sample is highly competitive. Unlike complex genomic sequencing or multiplexed proteomic assays—which often rely on expensive proprietary reagents and labor-intensive library preparations—our targeted metabolic ratio panel utilizes a streamlined extraction protocol and established internal standards. This high-throughput efficiency allows for the processing of hundreds of samples daily, potentially offering a more cost-effective alternative to existing specialized molecular diagnostics within healthcare systems. Nevertheless, broad clinical implementation faces several practical hurdles. The inherent technical complexity of mass spectrometry currently mandates a centralized laboratory model rather than a point-of-care application, requiring robust logistics for sample transport and specialized personnel for data interpretation. Furthermore, the diagnostic reliability of bipartite metabolic ratios is strictly contingent upon pre-analytical standardization. Because ratios such as L-acetylcarnitine/TML reflect dynamic metabolic fluxes associated with the tumoral “metabolic sink”, rigorous protocols for standardized blood collection, immediate plasma separation, and cold-chain maintenance are mandatory. Ensuring these conditions are met across diverse clinical settings remains a primary prerequisite for the successful translation of this high-precision diagnostic framework into routine urological practice.

## 5. Conclusions

In this study, we establish a discovery-phase proof-of-concept for a high-precision diagnostic liquid biopsy in prostate cancer, grounded in the physiological realities of systemic metabolic reprogramming. By leveraging a sensitive targeted-metabolomics platform, we identified a distinct plasma profile characterized by the profound depletion of TML and polyamine precursors, alongside a concomitant elevation in acylcarnitines and methylation markers. Supported by recent functional evidence characterizing the SLC25A45 mitochondrial transporter, these systemic shifts provide a measurable, functional readout of the “metabolic sink” hypothesis. Ultimately, the tumor’s voracious demand for fatty acid oxidation and androgen-driven polyamine synthesis creates a localized metabolic vacuum, driving the detectable depletion of critical circulating precursors.

Crucially, our findings demonstrate that translating these complex, interconnected networks into mathematically stable bipartite metabolic ratios—specifically L-acetylcarnitine/TML and sarcosine/putrescine—effectively mitigates systemic physiological noise while magnifying the underlying pathological signal. Subgroup analyses confirmed that these metabolic shifts are grade-independent, occurring consistently across the spectrum of tumor aggressiveness. Within our clinical cohort, a multivariable model utilizing this ratio-based approach achieved robust diagnostic separation (AUC = 0.99) across all clinical stages (I–IV), maintaining high sensitivity (AUC > 0.95) even in localized, low-grade disease. By serving as intrinsic normalizers, these metabolic ratios overcome the historical analytical limitations of single-metabolite biomarkers, demonstrating the clear potential to significantly improve upon the baseline predictive value of the current clinical standard, PSA.

In summary, this work highlights the clinical utility of targeting the fundamental bioenergetic bottlenecks of prostate cancer as a rational framework for biomarker discovery. While the current findings are exploratory and limited by the discovery-phase sample size, our data establishes a clear clinical use case for this platform as a frontline screening tool for early-stage disease. While large-scale, independent multi-center validation trials and dedicated in vivo functional models are necessary to confirm these findings, the integration of this targeted metabolomic signature into clinical workflows holds immense promise. Advancing this mechanistically grounded assay could significantly enhance diagnostic specificity, reduce the clinical and psychological burden of unnecessary biopsies, and ultimately facilitate more precise, personalized management strategies for patients with prostate cancer.

## Figures and Tables

**Table 1:** Demographic and clinical characteristics of prostate cancer patients in the current study (*n* = 35) *.

Parameter	
Age (years)	65 (56–74)
No history of chronic disease	10 (28%)
Hypertension	7 (20%)
Diabetes	9 (26%)
Ischemic heart disease	14 (40%)
History of smoking (active or ex-smoker)	17 (47%)
Tumor size (cm^3^)	45.3 (4.2–105.4)
Serum PSA level at diagnosis (ng/mL)	52.6 (4.0–319.7)
Gleason score	
≤7	14 (40%)
8–10	21 (60%)
Tumor stage	
Stage I	7 (20%)
Stage II	11 (31%)
Stage III	9 (26%)
Stage IV	8 (23%)

Abbreviation: PSA = prostate-specific antigen. * Data are shown as No. (%) or median (range). The metastatic status for the cohort is “Not Reported/Unknown”. No significant difference in age distribution was observed between the PCa and HC cohorts (Chi-square test, *p* = 0.963).

## Data Availability

All data supporting the findings of this study are available within the paper and its Supplementary Information. Individual-level metabolomics data and clinical annotations are available from the corresponding author upon reasonable request and execution of a data transfer agreement to protect patient privacy. Gene expression data from TCGA and other public datasets were obtained from the indicated repositories. Source data for all figures are provided. Complete360 platform protocols and calibration data are available from Complete Omics Inc. under appropriate licensing agreements.

## Supplementary Materials

The following supporting information can be downloaded at https://www.mdpi.com/article/10.3390/cancers18101571/s1, Figure S1: Diagnostic performance of the bipartite metabolite signature for PCa with high Gleason score (8–10, PCa_G-H, *n* = 21) vs. low Gleason score (≤7, PCa_G-L, *n* = 14). (A) L-Acetylcarnitine/TML Ratio: Box plots showing the relative levels based on the specific bipartite metabolite signature in human PCa_G-H vs. PCa_G-L plasma. ROC curve showing the corresponding diagnostic performance (AUC = 0.575; 95% CI, 0.384–0.793). (B) Sarcosine/Putrescine Ratio: Box plots showing the relative levels based on the specific bipartite metabolite signature in human PCa_G-H vs. PCa_G-L plasma. ROC curve showing the corresponding diagnostic performance (AUC = 0.498; 95% CI, 0.303–0.696). Figure S2: Diagnostic performance of the bipartite metabolite signature for PCa early detection [PCa with low Gleason score (≤7, PCa_G-L, *n* = 14) vs. HC (*n* = 35)]. (A) L-Acetylcarnitine/TML Ratio: Box plots showing the relative levels based on the specific bipartite metabolite signature in human PCa_G-L vs. HC plasma. ROC curve showing the corresponding diagnostic performance (AUC = 0.959; 95% CI, 0.882–0.998). (B) Sarcosine/Putrescine Ratio: Box plots showing the relative levels based on the specific bipartite metabolite signature in human PCa_G-L vs. HC plasma. ROC curve showing the corresponding diagnostic performance (AUC = 0.994; 95% CI, 0.969–1). Table S1: Differential metabolites between PCa and HC.

## Abbreviations

AR	androgen receptor
AUC	area under the curve
BPH	benign prostatic hyperplasia
CAP	College of American Pathologists
CI	confidence interval
CPT	carnitine palmitoyltransferase
FAO	fatty acid oxidation
HC	healthy normal controls
LC-MS	liquid chromatography–mass spectrometry
LC-MS/MS	liquid chromatography–tandem mass spectrometry
LDTs	laboratory-developed tests
MRM	multiple reaction monitoring
ODC1	ornithine decarboxylase 1
PCa	prostate cancer
PCA	principal component analysis
PLS-DA	partial least squares-discriminant analysis
PMID	PubMed identifier
PSA	primarily prostate-specific antigen
QqQ	triple quadrupole
ROC	receiver operating characteristic
SAM	S-adenosylmethionine
SDMA	symmetric dimethylarginine
SLC25A45	solute carrier family 25 member 45
SVM	support vector machine
TCGA	The Cancer Genome Atlas
TML	ε-N-trimethyl-L-lysine
UHPLC	ultra-high-performance liquid chromatography
UHPLC-QqQ-MS/MS	ultra-high-performance liquid chromatography coupled to triple quadrupole tandem mass spectrometry
VIP	variable importance in projection
