# Supplementary material for: The SLC25A45-TML Axis as a Biological Foundation for a Multivariable Plasma Metabolite Signature for High-Precision Prostate Cancer Detection
Source: Cancers (Basel). 2026 May 12;18(10):1571. doi: 10.3390/cancers18101571 (PMC13204762; doi:10.3390/cancers18101571)
Supplement: Supplementary file 1 [file cancers-18-01571-s001.zip › Figure S1_ROC of Gleason-subgroups.pdf]

A

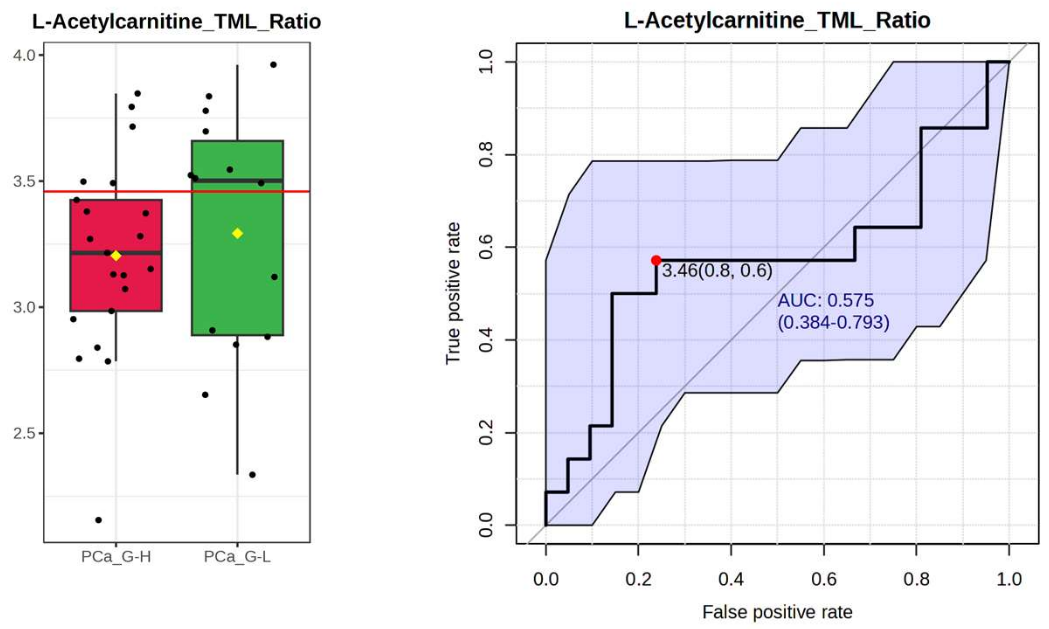

B

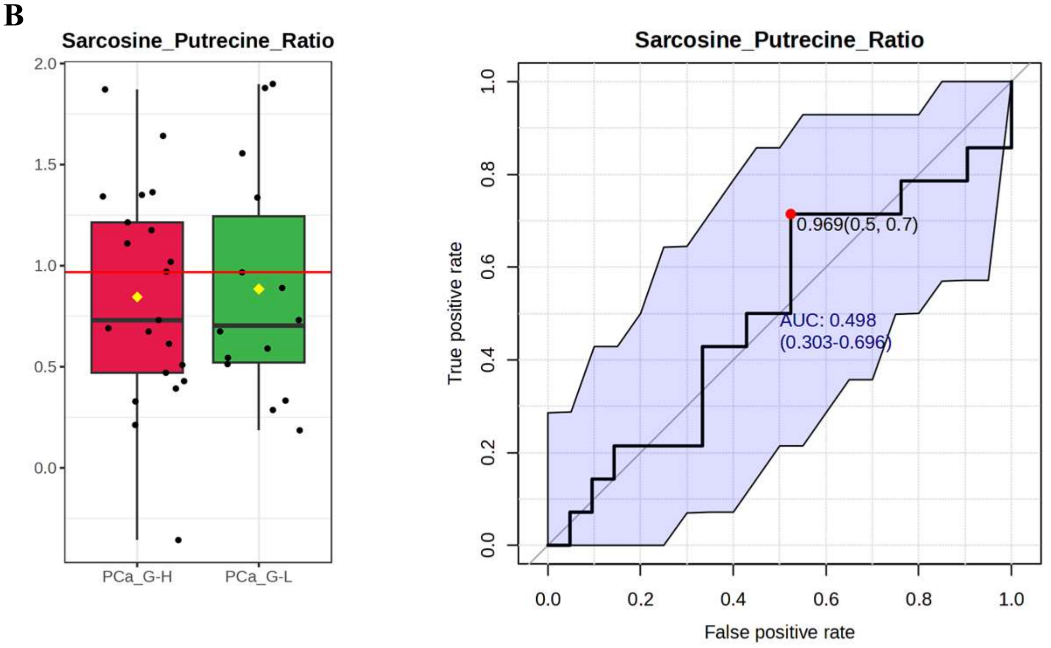

Figure S1. Diagnostic performance of the bipartite metabolite signature for PCa with high Gleason score (8-10, PCa_G-H, n=21) vs. low Gleason score (≤7, PCa_G-L, n=14). A, L-Acetylcarnitine/TML Ratio: Box plots showing the relative levels based on the specific bipartite metabolite signature in human PCa_G-H vs. PCa_G-L plasma. ROC curve showing the corresponding diagnostic performance (AUC=0.575; 95% CI, 0.384-0.793). **B,** Sarcosine/Putrescine Ratio: Box plots showing the relative levels based on the specific bipartite metabolite signature in human PCa_G-H vs. PCa_G-L plasma. ROC curve showing the corresponding diagnostic performance (AUC=0.498; 95% CI, 0.303-0.696).
