# Supplementary material for: The SLC25A45-TML Axis as a Biological Foundation for a Multivariable Plasma Metabolite Signature for High-Precision Prostate Cancer Detection
Source: Cancers (Basel). 2026 May 12;18(10):1571. doi: 10.3390/cancers18101571 (PMC13204762; doi:10.3390/cancers18101571)
Supplement: Supplementary file 1 [file cancers-18-01571-s001.zip › Figure S2_ROC of Gleason-Low vs HC.pdf]

**A**

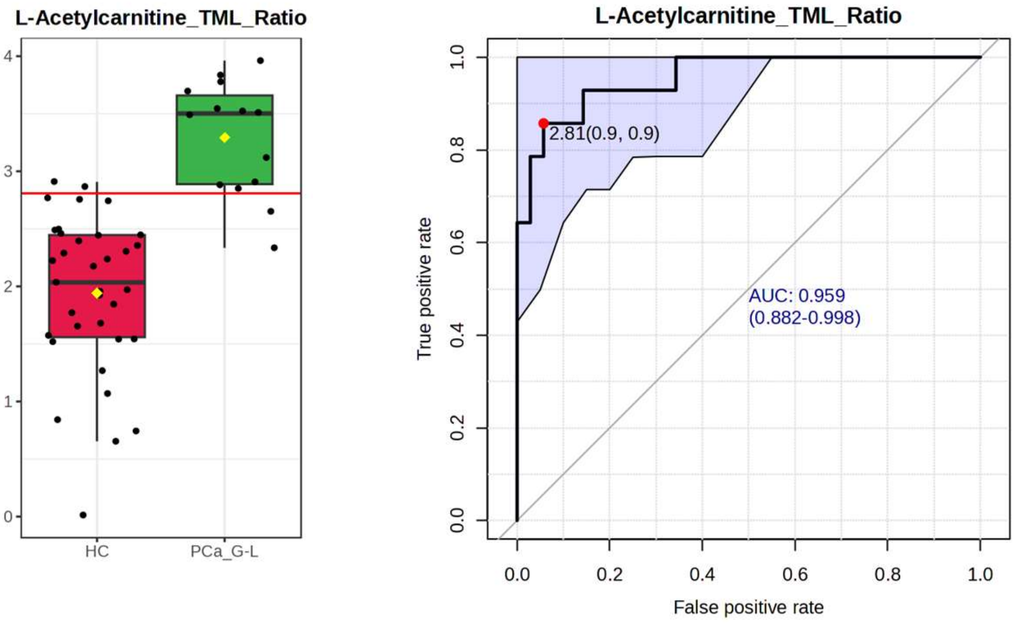


**B**

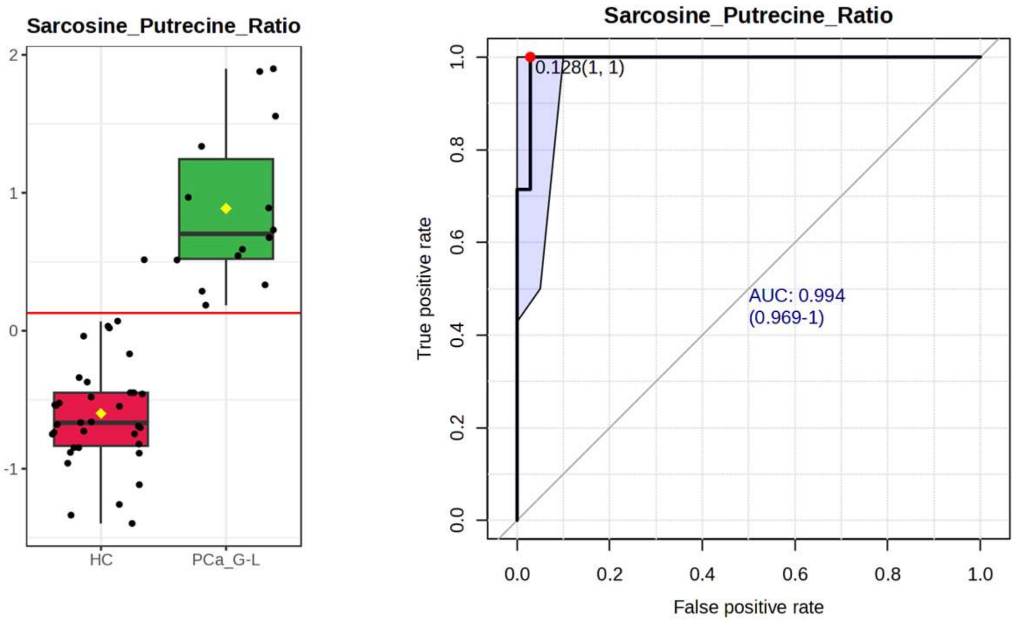


**Figure S2. Diagnostic performance of the bipartite metabolite signature for PCa early detection [PCa with low Gleason score (≤7, PCa_G-L, n=14) vs. HC (n=35)]. A,** L-Acetylcarnitine/TML Ratio: Box plots showing the relative levels based on the specific bipartite metabolite signature in human PCa_G-L vs. HC plasma. ROC curve showing the corresponding diagnostic performance (AUC=0.959; 95% CI, 0.882-0.998). **B,** Sarcosine/Putrescine Ratio: Box plots showing the relative levels based on the specific bipartite metabolite signature in human PCa_G-L vs. HC plasma. ROC curve showing the corresponding diagnostic performance (AUC=0.994; 95% CI, 0.969-1).
